# Forces exerted during microneurosurgery: a cadaver study

**DOI:** 10.1002/rcs.1568

**Published:** 2014-01-16

**Authors:** Hani J Marcus, Kourosh Zareinia, Liu Shi Gan, Fang Wei Yang, Sanju Lama, Guang-Zhong Yang, Garnette R Sutherland

**Affiliations:** 1 Hamlyn Centre, Imperial College LondonLondon, UK; 2 Department of Clinical Neurosciences, University of CalgaryAlberta, Canada

**Keywords:** neurosurgery, microsurgery, robotics, force

## Abstract

**Background:**

A prerequisite for the successful design and use of robots in neurosurgery is knowledge of the forces exerted by surgeons during neurosurgical procedures. The aim of the present cadaver study was to measure the surgical instrument forces exerted during microneurosurgery.

**Methods:**

An experimental apparatus was set up consisting of a platform for human cadaver brains, a Leica microscope to provide illumination and magnification, and a Quanser 6 Degrees-Of-Freedom Telepresence System for tissue manipulation and force measurements.

**Results:**

The measured forces varied significantly depending on the region of the brain (*P* = 0.016) and the maneuver performed (*P* < 0.0001). Moreover, blunt arachnoid dissection was associated with greater force exertion than sharp dissection (0.22 N vs. 0.03 N; *P* = 0.001).

**Conclusions:**

The forces necessary to manipulate brain tissue were surprisingly low and varied depending on the anatomical structure being manipulated, and the maneuver performed. Knowledge of such forces could well increase the safety of microsurgery. © 2014 The Authors. *The International Journal of Medical Robotics and Computer Assisted Surgery* published by John Wiley & Sons, Ltd.

## Introduction

The evolution of neurosurgery has been towards increasingly precise, delicate and safe surgical technique. Surgical robotics, which has the ability to eliminate tremor, and scale movements, has the potential to greatly enhance surgical performance. A prerequisite for the successful design and use of such robots is knowledge of the surgical instrument forces exerted by surgeons during neurosurgical procedures. The advent of surgical robotics may in turn allow, for the first time, the forces exerted during neurosurgical procedures to be routinely recorded 1–4. The corollary is that expert performances might be analyzed to determine the optimal force ranges utilized when performing robot-assisted neurosurgical procedures, providing quantitative feedback to trainees to further their development, and allowing for the possibility of force limits to be set to improve surgical safety 4.

Despite an abundance of anecdotal data on the instrument forces exerted during neurosurgical procedures, there remains a lack of quantitative data in the field. To date, the majority of studies addressing the forces necessary to manipulate brain tissue are either non-penetrating indentation studies designed to estimate the elastic properties of the brain 5–9, or penetration studies designed to estimate the forces required for probe insertion^10^–^13^. No studies have yet assessed the forces exerted during cranial microsurgery such as arachnoid dissection. To this end, the aim of the present study was to measure the surgical instrument forces exerted during robot-assisted microneurosurgery by performing a human cadaver study.

## Materials and methods

**Figure 1:**
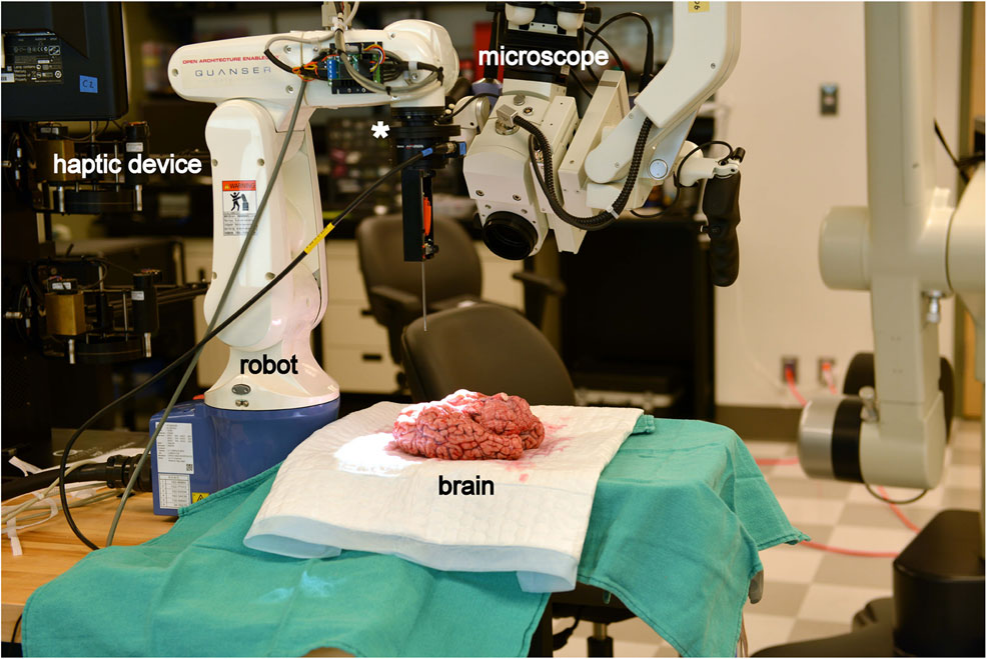
Experimental rig consisting of a platform for brain specimens, a Leica microscope, and a Quanser 6 DOF Teleoperation system. White star = location of the force/torque sensor between the DENSO Robot's end-effector and the tool-holder

An experimental apparatus was set up consisting of a platform for brain specimens, a Leica microscope (Leica Microsystems GmbH, Wetzlar, Germany) to provide illumination and magnification, and a Quanser 6 Degrees-Of-Freedom (DOF) Telepresence System (Quanser Inc, Ontario, Canada) for tissue manipulation and force measurements (Figure 1). The robotic master–slave setup included a DENSO VP Series 6-Axis Articulated Robot and the control module (DENSO Robotics, Aichi, Japan), Gamma Multi-Axis ATI Force/Torque Sensor (ATI Industrial Automation, North Carolina, USA) equipped with a 16-bit Data Acquisition Board (National Instruments, Texas, USA) for force/torque measurements, and a High-Definition Haptic Device (HD^2^) with the capability of providing 6 DOF force/torque feedback to the operator; this configuration allowed for continuous force feedback. The robot utilized an open-architecture interface with the QuaRC 2.2 (Quanser Real-time Control) Denso Robot block-set, alongside Simulink® (Mathworks, Massachusetts, USA), Matlab® (Mathworks, Massachusetts, USA) and Windows® (Microsoft, Washington, USA). The force/torque sensor was located between the DENSO Robot's end-effector and tool holder and measured 6 DOF force/torque with a capacity of 32 N and a resolution of 0.01 N (Figure 1). There was no force or position scaling involved and the ratio of force/torque feedback from the robot sensor to the haptic controller was set to 1:1. Calibration was independently verified using a Chatillon Digital Force Gauge (AMETEK, Florida, USA) with a capacity of 10 N and accuracy of 0.01 N.

Two fresh brain specimens were utilized for the study (Body Donation Program, Department of Anatomy, University of Calgary, Alberta, Canada). Brains were removed from their respective cranial cavities and dural sacs, leaving the brain, arachnoid and pia. In each specimen, a neurosurgical trainee (HJM) was asked to coagulate the brain surface using bipolar electrocautery on the 15 W setting and then perform a sequence of simple procedures: (1) a Beaver Mini-Blade was used to incise the coagulated area, (2) the Mini-Blade was used to carry the incision to a length of approximately 30 mm, and (3) a No 6 Rhoton spatula dissector with a 1.1 mm tip was inserted to a depth of approximately 5 mm and used to retract the brain approximately 5 mm. These maneuvers were carried out in the cerebrum (gyrus rectus, middle frontal gyrus, inferior temporal gyrus), cerebellum (hemispheres, vermis) and brainstem (midbrain, pons, medulla); and each was repeated twice on each side of the brain, unless it was too technically difficult to do so. In addition, the forces required to perform a corpus callosotomy and perforate the floor of the third ventricle were also measured.

The Beaver Mini-Blade and No 6 Rhoton dissector were also used to perform sharp and blunt dissection of the Circle of Willis respectively. An observer (KZ) carefully took note of iatrogenic injury to neurovascular structures during dissection (such as injury to tearing of small perforators). In cases of uncertainty, we recorded injury as having occurred.

Force vector data were produced each 0.2 ms (5 kHz). A Matlab® program was developed and used to extract data each 10 ms (100 Hz) and to record it in an Excel spreadsheet, and force vectors were summated. Statistical software (SPSS 20.0.0; IBM, New York, USA) was then used to calculate the median, interquartile range and maximum forces during individual procedures. The Mann–Whitney U test was used to compare the surgical instrument forces exerted in each brain specimen, and on the left- and right-side of the brain. The Kruskal–Wallis test was used to compare the forces exerted when performing different procedures, and between different brain regions; if significance was demonstrated post hoc analysis was performed using the Mann-Whitney U test, with the Bonferroni correction. The Mann–Whitney U test was then used to compare the forces exerted when performing sharp and blunt dissection, and the maneuvers that resulted in iatrogenic injury against those that did not. The threshold for significance was set at 5%.

## Results

In all, there was no significant difference in forces exerted between the two brain specimens, and between the left and right sides of the brains (*P* > 0.1). The median and interquartile ranges of forces exerted when performing different procedures in different brain regions are summarized in Table 1. The median force exerted when performing different procedures was significantly different (*P* < 0.0001); performing stab incisions (0.01 N) required significantly less force than carrying incisions (0.05 N; *P* < 0.0001) or retracting brain tissue (0.08 N; *P* < 0.0001). The median force exerted when manipulating different regions of the brain was also significantly different (*P* = 0.016); manipulating the brainstem (0.05 N) required significantly greater force than manipulating the cerebellum (0.02 N; *P* = 0.022) or cerebrum (0.03 N; *P* = 0.013).

**Table 1:** The median (interquartile range) of forces exerted (Newton) when performing simple procedures in different brain regions

		Median (interquartile range)		
		Stab Incision	Carrying Incision	Retraction
Cerebrum (n = 24)	Gyrus rectus (n = 8)	<0.01 (0.00 – 0.03)	0.02 (0.01 – 0.03)	0.03 (0.03 – 0.05)
	Inferior temporal gyrus (n = 8)	<0.01 (0.00 – 0.01)	0.02 (0.00 – 0.03)	0.07 (0.06 – 0.09)
	Middle frontal gyrus (n = 8)	<0.01 (0.00 – 0.01)	0.15 (0.12 – 0.18)	0.08 (0.06 – 0.10)
Cerebellum (n = 12)	Cerebellar hemisphere (n = 8)	0.01 (0.00 – 0.01)	0.03 (0.02 – 0.04)	0.08 (0.02 – 0.13)
	Cerebellar vermis (n = 4)	0.02 (0.01 – 0.02)	0.12 (0.12 – 0.12)	N.A.
Brainstem (n = 22)	Midbrain (n = 6)	0.01 (0.00 – 0.01)	0.11 (0.04 – 0.26)	0.15 (0.13 – 0.20)
	Pons (n = 8)	<0.01 (0.00 – 0.01)	0.05 (0.04 – 0.06)	0.18 (0.12 – 0.21)
	Medulla (n = 8)	0.01 (0.01 – 0.03)	0.09 (0.06 – 0.16)	0.09 (0.06 – 0.11)
Other (n = 8)	Corpus callosum (n = 4)	0.01 (0.00 – 0.03)	0.23 (0.09 – 0.43)	N.A.
	Perforating floor of third ventricle (n = 4)	<0.01 (0.00 – 0.01)	N.A.	N.A.

N.A. = Not applicable.

**Figure 2:**
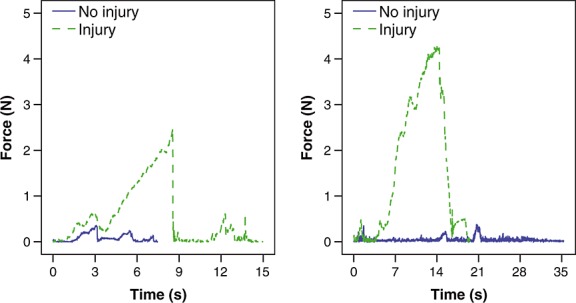
Illustrative examples of the forces exerted (Newton) over time when performing individual maneuvers, stratified according to whether they did or didn't result in iatrogenic injury, during (A) sharp dissection and (B) blunt dissection

**Figure 3:**
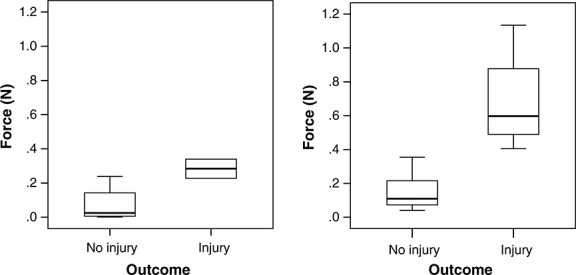
Box plot illustrating the median forces exerted (Newton) during (A) sharp and (B) blunt dissection around the Circle of Willis, stratified according to whether they did or didn't result in iatrogenic injury

Illustrative examples of the forces exerted over time during individual maneuvers are shown in Figure 2. The median, interquartile ranges, and maximum forces exerted when performing sharp and blunt arachnoid dissection around the Circle of Willis are summarized in Table 2 and Figure 3; maneuvers are stratified according to whether they did or didn't result in iatrogenic injury. The median force exerted during blunt dissection (0.22 N) and sharp dissection (0.03 N) was significantly different (*P* = 0.001). When performing sharp dissection the median force during maneuvers in which iatrogenic injury occurred (0.28 N) and those in which it did not (0.02 N) was significantly different (*P* = 0.011). When performing blunt dissection the median force during maneuvers in which iatrogenic injury occurred (0.60 N) and those in which it did not (0.11 N) was also significantly different (*P* = 0.004).

**Table 2:** The median (interquartile range) and maximum forces (Newton) exerted when performing sharp and blunt dissection of the Circle of Willis

		No injury (n = 34)	Injury (n = 6)
Sharp arachnoid dissection (n = 28)	Median (interquartile range)	0.02 (0.01 – 0.14)	0.28 (0.23 – 0.34)
	Maximum	1.33	2.49
Blunt arachnoid dissection (n = 12)	Median (interquartile range)	0.11 (0.07 – 0.22)	0.60 (0.49 – 0.88)
	Maximum	2.04	4.28

## Discussion

In this study the surgical instrument forces exerted during a number of robot-assisted procedures on fresh cadaver brains were successfully determined. The measured forces varied depending on the region of the brain and the maneuver performed. Significantly greater force was exerted when manipulating the brainstem (0.05 N) than the cerebellum (0.02 N; *P* = 0.022) or cerebrum (0.03 N; *P* = 0.013), and significantly less force was exerted when performing stab incisions (0.01 N) than carrying incisions (0.05 N; *P* < 0.0001) or retracting brain tissue (0.08 N; *P* < 0.0001). Moreover, blunt arachnoid dissection with a Rhoton No 6 dissector was associated with greater force exertion than sharp dissection with a Beaver Mini-Blade.

Existing literature on the forces exerted during neurosurgical procedures is sparse. Several studies have evaluated the mechanical properties of the brain itself through non-penetrating indentation experiments 5–9. As early as 1976, Walsh *et al*. measured the elastic response of brain tissue in dogs using a diaphragm-type pressure sensor mounted co-linear with a displacement transducer, and described a nonlinear response 5. Miller *et al*. conducted a study on an exposed porcine brain and reported that approximately 0.3 N was required for up to 4 mm of brain displacement, slightly greater than predicted by their hyper-viscoelastic constitutive model 7. Gefen *et al*. performed a similar non-penetrating indentation study on exposed porcine brains, comparing the forces required to deform brain tissue during *in vivo*, *in situ* and *in vitro* experiments, and demonstrating that most of the brain's mechanical properties are unaffected by perfusion pressure, and can therefore be examined *in vitro* 9. These findings are broadly comparable with those of the present study; we estimated that the median forces to retract brain tissue 5 mm varied from 0.03 N in the cerebrum to 0.18 N in the brainstem.

Alongside the aforementioned studies evaluating the mechanical properties of the brain, a number of studies have utilized penetration experiments to estimate the forces required to insert probes of various characteristics into neural tissue. Howard *et al*. measured the penetration forces on 2.5 mm spheres and the drag forces on a 3 mm ventricular catheter advanced 20–30 mm deep into the brain tissue of patients undergoing temporal lobectomy, and reported forces of 0.08 N and 0.03 N respectively 11. Chen *et al*. measured the forces exerted when placing a 3 mm ventricular catheter into a porcine brain during validation of an agarose gel phantom, and reported a typical penetration force of less than 0.1 N 13. Sharp *et al*. addressed micrometer-scale penetration dynamics, assessing the influence of probe size and geometry, and also reported that low forces were required 10. When utilizing a 200 µm diameter flat punch probe, for example, a maximum force of 0.03 N was exerted. In the current study, the forces exerted to penetrate brain tissue were very low, varying from <0.01 N to 0.02 N, although greater forces were required to extend the incision. We speculate that the very low forces observed in our study are the result of its distinct methodology; to better reflect standard operative neurosurgical practice the brain surface was coagulated with bipolar electrocautery prior to any maneuvers, and a sharp beaver mini-blade instrument used to incise the brain.

Cranial microsurgery, such as aneurysm clipping or tumor resection, is characterized by precise and delicate complex maneuvers such as dissection around critical neurovascular structures, rather than simple brain tissue retraction and penetration. To date, no previous studies have reported the forces actually exerted during such neurosurgical procedures. In other surgical disciplines, the forces exerted during, for example, laparoscopic cholecystectomy was reported to be approximately 17 N 14. In comparison, the instrument forces measured during sharp and blunt arachnoid dissection were an order of magnitude less, and with median forces typically less than 1 N.

### Limitations

The present study has several limitations. First, although forces were measured carefully using an externally calibrated system, the penetration forces detected were surprisingly low compared with other studies in the literature, with several readings falling below the 0.01 N resolution of the experimental configuration. Nonetheless, the fact that such low forces were observed was in itself an important finding, and other readings such as microsurgical dissection were well within the operating range of the force sensor. Second, the cadaver brains utilized undoubtedly had different tissue properties to living brain tissue. While animal studies might have allowed for *in vivo* measurements, the gross size and structure of, for example, porcine brains differ considerably from their human counterparts. Fresh cadaver brains were used to ameliorate this as they are anatomically accurate and previous studies have suggested that there is in fact little difference in the mechanical properties of living and cadaver brain tissues 9. Third, although arachnoid dissection with a Beaver Mini-Blade or No 6 Rhoton dissector is almost certainly a far better reflection of *in vivo* microneurosurgery than previous studies, it still does not reflect the full spectrum of instruments available to neurosurgeons, the complex and varied technical maneuvers of actual neurosurgical operations, and the range of speeds with which such maneuvers may be performed. Procedures such as microanastomosis, for example, may utilize a considerably different range of forces. Fourth, the maneuvers were performed by a single surgical trainee. While the forces exerted during simple procedures such as retraction are likely to be relatively independent of the operator, the surgical forces exerted during microsurgery are known to vary and are greater with novices than experts 15, and the forces applied by the neurosurgical trainee in this study are therefore likely to be higher than if experienced consultants had performed it. Although not explored in the present study, additional factors such as surgeon fatigue and perhaps the nature of the case (e.g. emergency craniotomy) could also influence technical performance, and therefore forces exerted 16.

### Generalizability and concluding remarks

The present study suggests that the instrument forces exerted during microsurgical arachnoid dissection are relatively low, with median forces less than 1 N, and maximum forces less than 5 N. Nonetheless, *in vivo* forces are likely to vary depending on a number of factors including the patient's particular brain characteristics, the nature of their pathology (such as tumor consistency), the experience of the operating surgeon, and the surgical procedure performed. In the future, widespread adoption of surgical robots into mainstream practice might allow for quantification of surgical forces on a routine basis, enhancing not only the precision, accuracy, and safety of cranial microsurgery, but also providing valuable feedback to neurosurgical trainees.

## Previous presentation

Interim findings of this study were presented at the Hamlyn Symposium on Medical Robotics 2013. Otherwise, the authors confirm no prior or duplicate publication or submission elsewhere of any part of this work.

## References

[b1] Sutherland GR, Wolfsberger S, Lama S, Zarei-nia K (2013). The evolution of neuroArm. Neurosurgery.

[b2] Marcus H, Nandi D, Darzi A, Yang GZ (2013). Surgical robotics through a keyhole: from today's translational barriers to tomorrow's “disappearing” robots. IEEE Trans Biomed Eng.

[b3] Sutherland GR, Lama S, Gan LS, Wolfsberger S, Zareinia K (2013). Merging machines with microsurgery: clinical experience with neuroArm. J Neurosurg.

[b4] Lang MJ, Sutherland GR (2010). Informatic surgery: the union of surgeon and machine. World Neurosurg.

[b5] Walsh EK, Schettini A (1976). Elastic behavior of brain-tissue in vivo. Am J Physiol.

[b6] Walsh EK, Furniss WW, Schettini A (1977). Measurement of brain elastic response in vivo. Am J Physiol.

[b7] Miller K, Chinzei K, Orssengo G, Bednarz P (2000). Mechanical properties of brain tissue in-vivo: experiment and computer simulation. J Biomech.

[b8] Gefen A, Gefen N, Zhu QL, Raghupathi R, Margulies SS (2003). Age-dependent changes in material properties of the brain and braincase of the rat. J Neurotrauma.

[b9] Gefen A, Margulies SS (2004). Are in vivo and in situ brain tissues mechanically similar?. J Biomech.

[b10] Sharp AA, Ortega AM, Restrepo D, Curran-Everett D, Gall K (2009). In vivo penetration mechanics and mechanical properties of mouse brain tissue at micrometer scales. IEEE Trans Biomed Eng.

[b11] Howard MA, Abkes BA, Ollendieck MC, Noh MD, Ritter RC, Gillies GT (1999). Measurement of the force required to move a neurosurgical probe through in vivo human brain tissue. IEEE Trans Biomed Eng.

[b12] Ritter RC, Quate EG, Gillies GT, Grady MS, Howard MA, Broaddus WC (1998). Measurement of friction on straight catheters in in vitro brain and phantom material. IEEE Trans Biomed Eng.

[b13] Chen ZJ, Gillies GT, Broaddus WC (2004). A realistic brain tissue phantom for intraparenchymal infusion studies. J Neurosurg.

[b14] Hwang H, Lim J, Kinnaird C (2006). Correlating motor performance with surgical error in laparoscopic cholecystectomy. Surg Endosc.

[b15] Harada K, Minakawa Y, Baek Y (2011). Microsurgical skill assessment: toward skill-based surgical robotic control. Conf Proc IEEE Eng Med Biol Soc.

[b16] Eastridge BJ, Hamilton EC, O'Keefe GE (2003). Effect of sleep deprivation on the performance of simulated laparoscopic surgical skill. Am J Surg.

